# Bursectomy and non-bursectomy D2 gastrectomy for advanced gastric cancer, initial experience from a single institution in China

**DOI:** 10.1186/s12957-015-0744-x

**Published:** 2015-12-08

**Authors:** Wei-Han Zhang, Xin-Zu Chen, Kun Yang, Kai Liu, Zhi-Xin Chen, Bo Zhang, Zong-Guang Zhou, Jian-Kun Hu

**Affiliations:** Department of Gastrointestinal Surgery, West China Hospital, Sichuan University, Chengdu, Sichuan Province China; Laboratory of Gastric Cancer, State Key Laboratory of Biotherapy, West China Hospital, Sichuan University, Chengdu, Sichuan Province China

**Keywords:** Gastric cancer, Gastrectomy, Bursectomy, Complication, Prognosis

## Abstract

**Background:**

The aim of this study is to evaluate the safety and efficacy of bursectomy of D2 gastrectomy in terms of postoperative complications and short-term survival outcomes.

**Methods:**

From January 2012 to December 2013, data of 406 gastric cancer patients with advanced tumor stages and who underwent D2 radical gastrectomy and were grouped according to whether bursectomy was performed or not in West China Hospital, Sichuan University, were analyzed.

**Results:**

Finally, 159 patients were in bursectomy group and 247 patients in non-bursectomy group. Surgical duration was 260.1 ± 43.4 min in the bursectomy group, compared to 227.9 ± 48.6 min in the non-bursectomy group (*p* < 0.001). The intraoperative blood loss was comparable between the bursectomy group and the non-bursectomy group (198.9 ± 63.5 vs. 201.1 ± 53.7 ml, *p* = 0.729). Postoperative morbidity rate showed no significant difference between the two groups, which were 23.3 % in the bursectomy group and 17.8 % in the non-bursectomy group, *p* = 0.179. The overall survival outcomes of patients were compared between the two groups of all patients (*p* = 0.055): patients who underwent distal gastrectomy (*p* = 0.129) and total gastrectomy (*p* = 0.016) and pT2-3 stage patients (*p* = 0.117) and pT4a stage patients (*p* = 0.128). The multivariate survival analysis identified that bursectomy or not, pT stage and pN stage were independent prognostic risk factors for the overall survival.

**Conclusions:**

The bursectomy might increase the surgical duration when the D2 gastrectomy was done. Experienced surgeons can perform it safely. However, for the survival benefits of bursectomy, long-term, large sample sized, and high-quality randomized controlled trials are expected.

**Electronic supplementary material:**

The online version of this article (doi:10.1186/s12957-015-0744-x) contains supplementary material, which is available to authorized users.

## Background

Gastric cancer is the second most common cause of cancer-related death due to its malignant potential [1–3]. Owing to the absence of large sample size prospective randomized control trials with long-term survival results, there have been many disputes on the clinical use of bursectomy with gastrectomy for advanced gastric cancer patients. Bursectomy is mainly defined as a complete dissection of the peritoneal lining covering the pancreas and the anterior plane of the transverse mesocolon and with an omentectomy during gastrectomy [4, 5]. This surgical technique has been developed as a part of radical gastrectomy with the aim of removing the potential microscopic tumor seeding since the 1960s in Japan and based on the following oncological and anatomical theories [6–8]: (1) prevents peritoneal recurrences by eliminating micro-metastatic disease in the lesser sac of peritoneal cavity and (2) complete resection of the subpyloric lymph nodes (LNs). However, the therapeutic value of bursectomy is controversial because the survival benefit is uncertain. One randomized controlled trial performed by the Osaka University Clinical Research Group found that bursectomy may improve the survival outcomes in pT3-4 stages patients and should not be dropped [9]. However, other two retrospective studies indicated that there were no survival benefits for bursectomy when compared with non-bursectomy for gastric cancer patients [10, 11]. According to a recently meta-analysis that included one randomized controlled trial (RCT) and three non-RCTs, Shen et al. found that there was no survival benefits for the bursectomy when compared with non-bursectomy surgery for gastric cancer patients and the bursectomy was not recommended as a routinely procedure for gastric cancer surgery [12]. Meanwhile, the treatment guideline of Japanese Gastric Cancer Association (JGCA) only recommended that bursectomy can be selectively used according to the specific tumor stage and location [13, 14].

Due to the high proportion of advanced stage patients in China, we analyzed the clinicopathological data, the postoperative complications and survival outcomes for advanced gastric cancer patients who underwent D2 radical distal and total gastrectomy with or without bursectomy to report the initial experience of our center.

## Methods

### Ethical statement

The Ethics Committee of West China Hospital, Sichuan University, approved this retrospective study. The participants’ written consent were not obtained, but the patients’ records were anonymous to analysis.

### Patients

From January 2012 to December 2013, those gastric cancer patients from the Department of Gastrointestinal Surgery, West China Hospital, Sichuan University, were included in this study based on the following criteria: (1) histologically confirmed gastric adenocarcinoma; (2) pT2-4, N0-3, and M0 stages according to the Japanese Classification of Gastric Carcinoma [15]; (3) distal gastrectomy and total gastrectomy by the conventional open method; (4) D2 lymphadenectomy according to the Japanese gastric cancer treatment guidelines [13]; and (5) curative resection without residual tumors (R0 resection). And those patients with distant metastases or positive cytology examination were excluded. Those patients underwent preoperative adjuvant chemotherapy were excluded to minimize the survival influence of it. Finally, the records of the patients who fulfilled the inclusion and exclusion criteria for this study were obtained and analyzed.

### Bursectomy and operative procedure

**Fig. 1:**
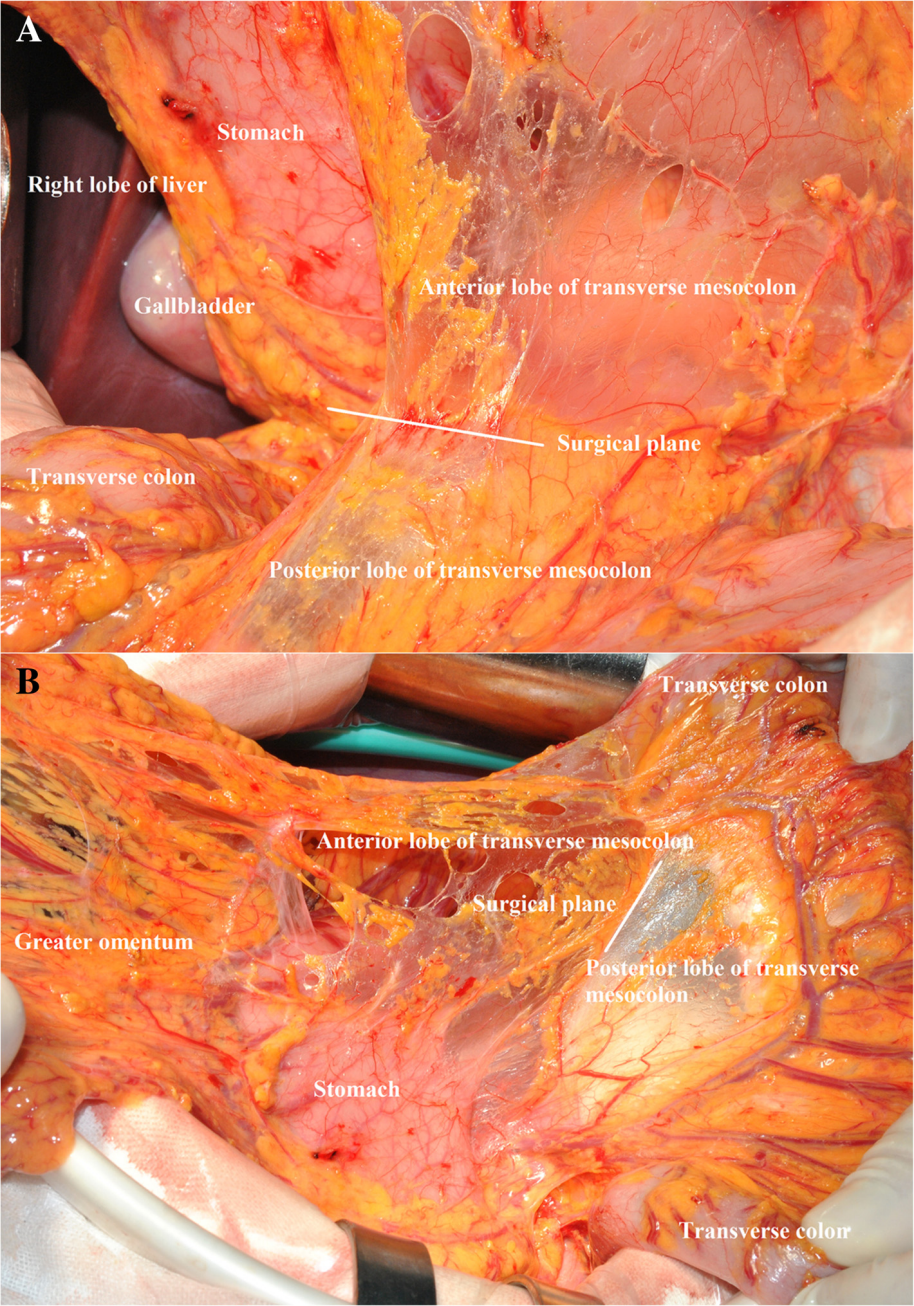
Removal of the anterior lobe of the transverse mesocolon. **a** The surgical plane of the removal of the anterior lobe of the transverse mesocolon (*front view*). **b** The surgical plane of the removal of the anterior lobe of the transverse mesocolon (*side view*)

**Fig. 2:**
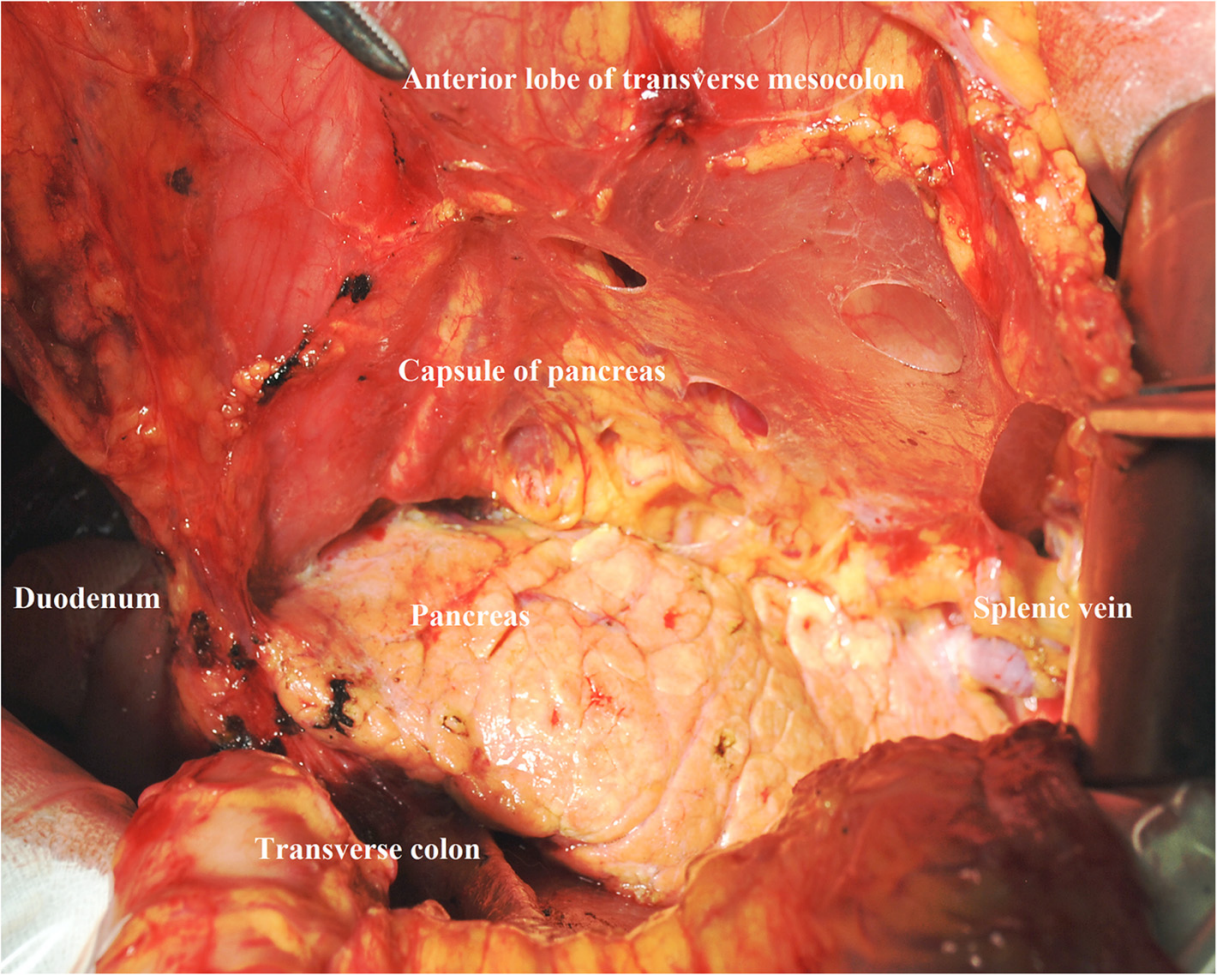
Complete removal of the capsule of the pancreas and the anterior lobe of the transverse mesocolon

All the gastric cancer patients included in this study had undergone surgical treatments by well-trained surgeons in the gastric cancer treatment team of our department. Because this is not a randomized controlled study, patients who underwent bursectomy or non-bursectomy surgery were intraoperatively decided by the chief surgeons. The surgical treatment principle was adopted by the Japanese Gastric Cancer Treatment Guidelines which was published by the JGCA [16]. For the resection patterns, those advanced gastric cancer patients with tumors located in the upper or middle third had total gastrectomy. Patients with tumors located in the lower third of the stomach and with lymph nodes metastasis in no. 1, no. 2, and no. 4sb stations during the intraoperative frozen section evaluation also underwent total gastrectomy. Only lower third gastric cancers and distal gastrectomy can secure the tumor-free radical resection and have distal gastrectomy. According to the guidelines of the JGCA, in order to attain the complete dissection of subpyloric lymph nodes, the right side of the anterior plane of the transverse mesocolon and the pancreas was routinely resected, which was partially bursectomy. Besides the right side of the bursa omentalis, total bursectomy should be with en bloc resection of the peritoneal lining of the bursa omentalis (the anterior lobe of the transverse mesocolon and the pancreas), and the anterior lobe of the transverse mesocolon and the capsule of the pancreas should be dissected as much as possible [10], which was defined as the total bursectomy. Patients who underwent partial bursectomy (right side) were included in the non-bursectomy group, and patients who underwent total bursectomy were grouped in the bursectomy group. During the surgical procedures, the exposure of the surgical field, appropriate tension is essential to the success of completely removing the anterior lobe of the transverse mesocolon and the capsule of the pancreas (Fig. 1). Therefore, when the bursectomy is finished, only the posterior layer of the transverse mesocolon remained and the anterior lobe of the transverse mesocolon and the capsule of the pancreas were entirely separated from the transverse mesocolon and the pancreas (Fig. 2). Additional surgical procedures between the two groups were similar. D2 lymphadenectomy was carried out according to treatment guidelines published by Japanese Gastric Cancer Association [13]. Roux-en-Y esophagojejunostomy reconstructions were for total gastrectomy. And for distal gastrectomy, Billroth type I/II or Roux-en-Y gastrojejunostomy reconstructions were performed according to the tumor characteristics and others.

### Clinicopathological characteristics

Clinicopathological characteristics, perioperative morbidity, and mortality were analyzed. The cross-sectional location and the longitudinal location of tumors were recorded according to the standard of the Japanese Classification of Gastric Carcinoma [15]. Lymph nodes were separately examined by the anatomical definitions of lymph node stations, according to the Japanese guidelines [13]. The number of positive and examined lymph nodes of the no. 4d, no. 4sb, and no. 6 LNs were compared between the two groups. The cancers were staged according to the Union for International Cancer Control (UICC) tumor-node-metastasis (TNM) system, 7th Edition [17]. Postoperative mortality and morbidity were counted for 30 days or during the same hospitalization. Specifically, the classification of pancreatic fistula was according to the international study group of pancreatic fistula classification [18]. The grades of the postoperative complications by the Clavien–Dindo classification of surgical complications between the two groups were evaluated and compared [19].

### Follow-up

The follow-up was performed by means of routinely outpatient visit. Mail and telephone interviews were the supplementary methods. The follow-up information was updated until January 1, 2015. The follow-up rate, median follow-up duration (months), recurrence type, and overall survival outcomes were analyzed in the study. Recurrence types include the local-regional recurrence (anastomotic recurrence) type, peritoneal seeding recurrence type, hematogenous recurrence (liver, pulmonary, bone, et al.) type, distal lymph nodes (non-regional lymph nodes) type, and multisite recurrence type. The reasons for the patients lost to follow-up were predominantly refusal of the outpatient visit or a change in the telephone number and address.

### Statistics

Statistical analysis was conducted using SPSS statistics software, version 19.0 (SPSS, Chicago, Illinois, USA). For the continuous variables with normal distribution, they were analyzed by the one-way ANOVA test. Both the continuous variables without normal distribution and the ranked variables were weighed by the Mann–Whitney *U* test. The categorical variables were adopted with the Pearson’s chi-square test (or likelihood ratio). Survival outcomes were reported to Kaplan–Merrier method, log-rank test. Multivariate adjusted factor survival analysis was performed using Cox proportional hazard modeling. Hazard ratio (HR) and 95 % confidential interval (CI) were used to present the results of the univariate and multivariate survival analysis. Two-tailed *p* value of less than 0.05 was regarded as statistically significant.

## Results

### Clinicopathological features

From January 2012 to December 2013, 406 gastric cancer patients were included in the final analysis, 159 of whom in bursectomy group and 247 in non-bursectomy group. Most of the clinicopathological characteristics were comparable between the two groups, such as age, gender, tumor size, and tumor locations, and as well as tumor stages (Tables 1 and 2). Surgical duration was significantly increased in the bursectomy group when compared with the non-bursectomy group (260.1 ± 43.4 vs. 227.9 ± 48.6 min, *p* < 0.001, respectively). However, the amount of blood loss was comparable between bursectomy group than the non-bursectomy group (198.9 ± 43.4 vs. 201.1 ± 53.7 ml, *p* = 0.729). Meanwhile, we found out that more proportion of patients underwent total gastrectomy in bursectomy group than non-bursectomy group (54.1 vs. 25.5 %, *p* < 0.001). Also, there existed difference in the longitudinal location of tumors (upper, middle, lower) between the bursectomy and non-bursectomy groups (39.0, 13.8, 47.2 % vs. 11.2, 18.2, 70.4 %, respectively, *p* < 0.001).

**Table 1:** Clinicopathological characteristics of the non-Bursectomy group and the bursectomy group

Characteristics	Non-bursectomy group	Bursectomy group	*p* value
	*N* = 247 (%)	*N* = 159 (%)	
Age (years)	58.3 ± 12.2	57.3 ± 11.5	0.385
Gender			0.513
Male	160 (64.8)	108 (67.9)	
Female	87 (35.2	51 (32.1)	
Cross-sectional location			0.008
Lesser	124 (50.2)	101 (63.5)	
Greater	15 (6.1)	11 (6.9)	
Anterior	34 (13.8)	8 (5.0)	
Posterior	40 (16.2)	15 (9.4)	
Circumferential involvement	34 (13.8)	24 (15.1)	
Longitudinal location			<0.001
U	28 (11.2)	62 (39.0)	
M	45 (18.2)	22 (13.8)	
L	174 (70.4)	75 (47.2)	
Differentiation grade			0.328
Well-moderate	29 (11.7)	24 (15.1)	
Poor-undifferentiated	218 (88.3)	135 (84.9)	
Macroscopic type			0.307
Type 1	14 (5.7)	4 (2.5)	
Type 2	99 (40.1)	75 (47.2)	
Type 3	118 (47.8)	71 (44.7)	
Type 4	16 (6.5)	9 (5.7)	
Tumor size (cm)	5.7 ± 2.4	5.6 ± 2.3	0.537
Surgical duration (min)	227.9 ± 48.6	260.1 ± 43.4	<0.001
Blood loss (ml)	201.1 ± 53.7	198.9 ± 63.5	0.729
Resection patterns			<0.001
DG	184 (74.5)	73 (45.9)	
TG	63 (25.5)	86 (54.1)	

*Abbreviations*: *U* upper, *M* middle, *L* lower, *DG* distal gastrectomy, *TG* total gastrectomy

**Table 2:** Lymph node status of the non-bursectomy group and the bursectomy group

Characteristics	Non-bursectomy group	Bursectomy group	*p* value
	*N* = 247 (%)	*N* = 159 (%)	
T stage			0.136
T2	48 (19.4)	20 (12.6)	
T3	45 (18.2)	37 (23.3)	
T4	154 (62.3)	102 (64.2)	
N stage			0.593
N0	60 (24.3)	38 (23.9)	
N1	51 (20.6)	25 (15.7)	
N2	43 (17.4)	28 (17.6)	
N3	93 (37.7)	68 (42.8)	
TNM stage			0.724
Ib	17 (6.9)	8 (5.0)	
IIa	24 (9.7)	16 (10.1)	
IIb	45 (18.2)	30 (18.9)	
IIIa	47 (19.0)	22 (13.8)	
IIIb	42 (17.0)	30 (18.9)	
IIIc	72 (29.1)	53 (33.3)	
No.4D LNs			
Number of positive	0.7 ± 1.3	0.8 ± 1.8	0.675
Number of examined	3.7 ± 2.7	4.9 ± 4.0	0.001
No.4SB LNs			
Number of positive	0.1 ± 0.5	0.1 ± 0.8	0.617
Number of examined	1.7 ± 2.2	1.6 ± 2.3	0.743
No.6 LNs			
Number of positive	1.2 ± 2.0	0.9 ± 1.7	0.158
Number of examined	4.4 ± 3.4	4.8 ± 3.8	0.362
Total			
Number of positive	5.9 ± 6.4	7.5 ± 8.7	0.045
Number of examined	25.4 ± 9.9	40.6 ± 17.5	<0.001

*LNs* lymph nodes

### Lymph node status

Total number of examined LNs was significantly higher in bursectomy group than non-bursectomy group (40.6 ± 17.5 vs. 25.4 ± 9.9, *p* < 0.001). Besides, the number of positive lymph nodes was also higher in the bursectomy group than the non-bursectomy group (7.5 ± 8.7 vs. 5.9 ± 6.4, *p* = 0.045). Comparison of the number of the positive LNs and the number of examined LNs between the two groups in no. 4d LNs, no. 4sb LNs, and no. 6 LNs were listed in Table 2. The number of examined LNs was 4.9 ± 4.0 in no. 4d LNs of bursectomy group compared to 3.7 ± 2.7 of non-bursectomy group, *p* < 0.001. The number of examined LNs in no. 4SB LN and no. 6 LNs was similar between the two groups, *p* = 0.743 and *p* = 0.362, respectively.

### Mortality and morbidity

There was no incidence of intraoperative mortality of patients in this study. The mean postoperative hospital stay was 11.4 ± 4.4 days in bursectomy group and 11.4 ± 4.4 days in non-bursectomy group (*p* = 0.850). Postoperative morbidity rate was comparable between the bursectomy and the non-bursectomy groups (26.4 vs. 17.8 %, *p* = 0.179). Details of postoperative complications were listed in Table 3. Reoperation was needed in five patients within 30 days of operation: three patients for abdominal cavity hemorrhage and two patients for intra-abdominal abscesses in bursectomy group and two patients for abdominal cavity hemorrhage and another patient of intestinal obstruction in non-bursectomy group. There was just one hospital death; a patient in non-bursectomy group died of postoperative acute myocardial infarction. All patients recovered well and successfully discharged from the hospital, including one patient in bursectomy group and one patient in non-bursectomy group who had a duodenal stump fistula with full drainage and effective nutrition support with more than 30-day postoperative hospital stay. We also compared the grades of postoperative complications according to the Clavien–Dindo classification and found that it was comparable between the two groups, *p* = 0.783 [19].

**Table 3:** Short-term outcomes of the non-bursectomy group and the bursectomy group

	Non-bursectomy group	Bursectomy group	*p* value
	*N* = 247 (%)	*N* = 159 (%)	
Postoperative hospital stay (days)	11.4 ± 4.4	11.4 ± 4.4	0.850
Overall morbidity	44 (17.8)	37 (23.3)	0.179
PPCs	19 (43.18)	15 (40.5)	
Pancreatic fistula	4 (9.1)	5 (13.5)	
Gastroparesis	5 (11.4)	3 (8.1)	
Intraperitoneal hemorrhage	3 (6.8)	5 (13.5)	
Anastomotic leakage	1 (2.3)	1 (2.7)	
SSIs	3 (6.8)	1 (2.7)	
Intraperitoneal infection	5 (11.4)	6 (16.2)	
Postoperative ileus	3 (6.8)	1 (2.7)	
Acute myocardial infarction	1 (2.3)	0 (0)	
Postoperative mortality			
Classification of complications^a^			0.759
Grade I	21 (47.7)	16 (43.2)	
Grade II	19 (43.2)	16 (43.2)	
Grade III	2 (4.5)	3 (8.1)	
Grade IV	1 (2.3)	2 (5.4)	
Grade V	1 (2.3)	0 (0)	

*Abbreviations*: *PPCs* postoperative pulmonary complications, *SSIs*, surgical site infections

^a^The Clavien–Dindo classification of surgical complications

### Survival outcomes

**Fig. 3:**
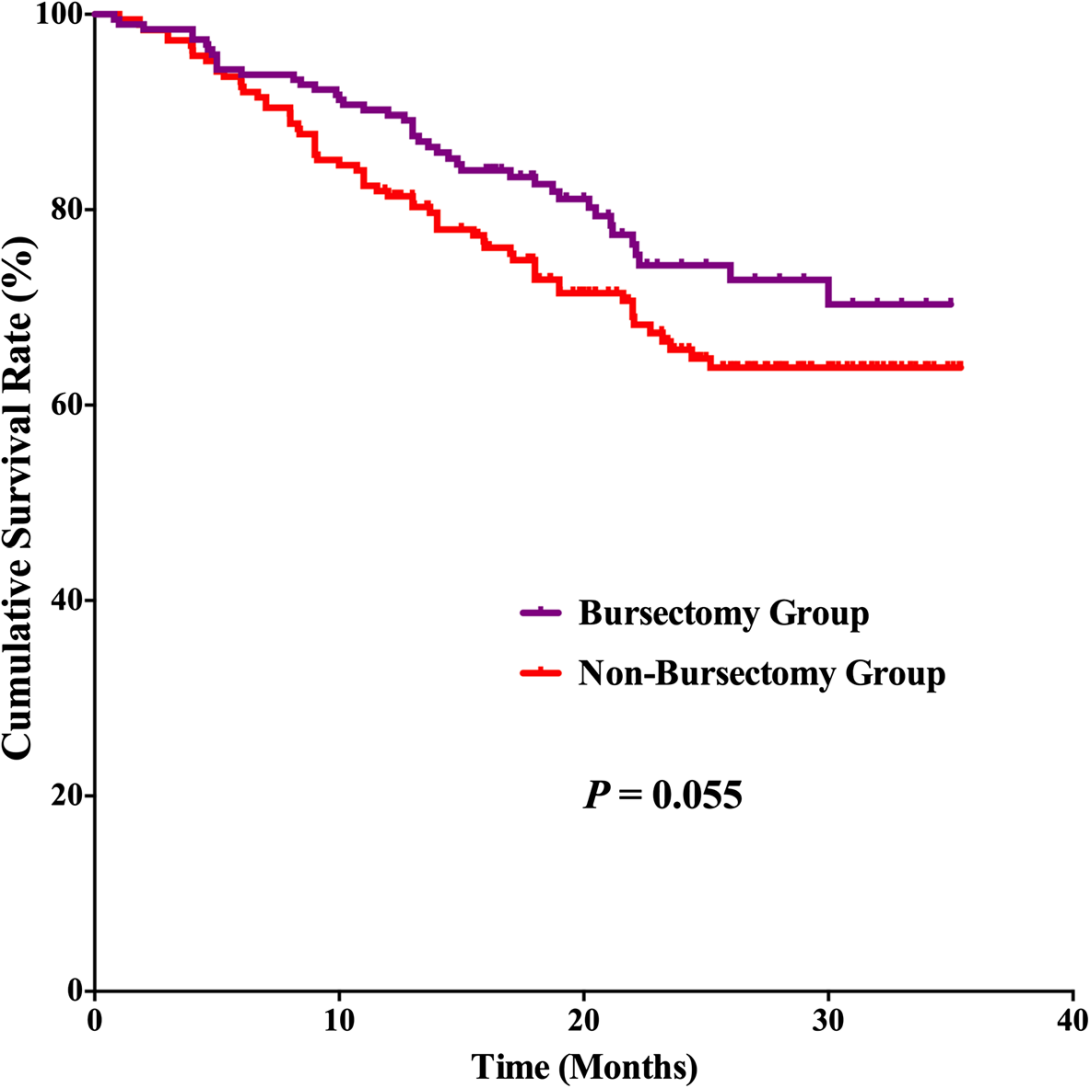
Survival curves among the bursectomy group and non-bursectomy group. There was no significant difference in survival between the two groups (*p* = 0.055)

**Fig. 4:**
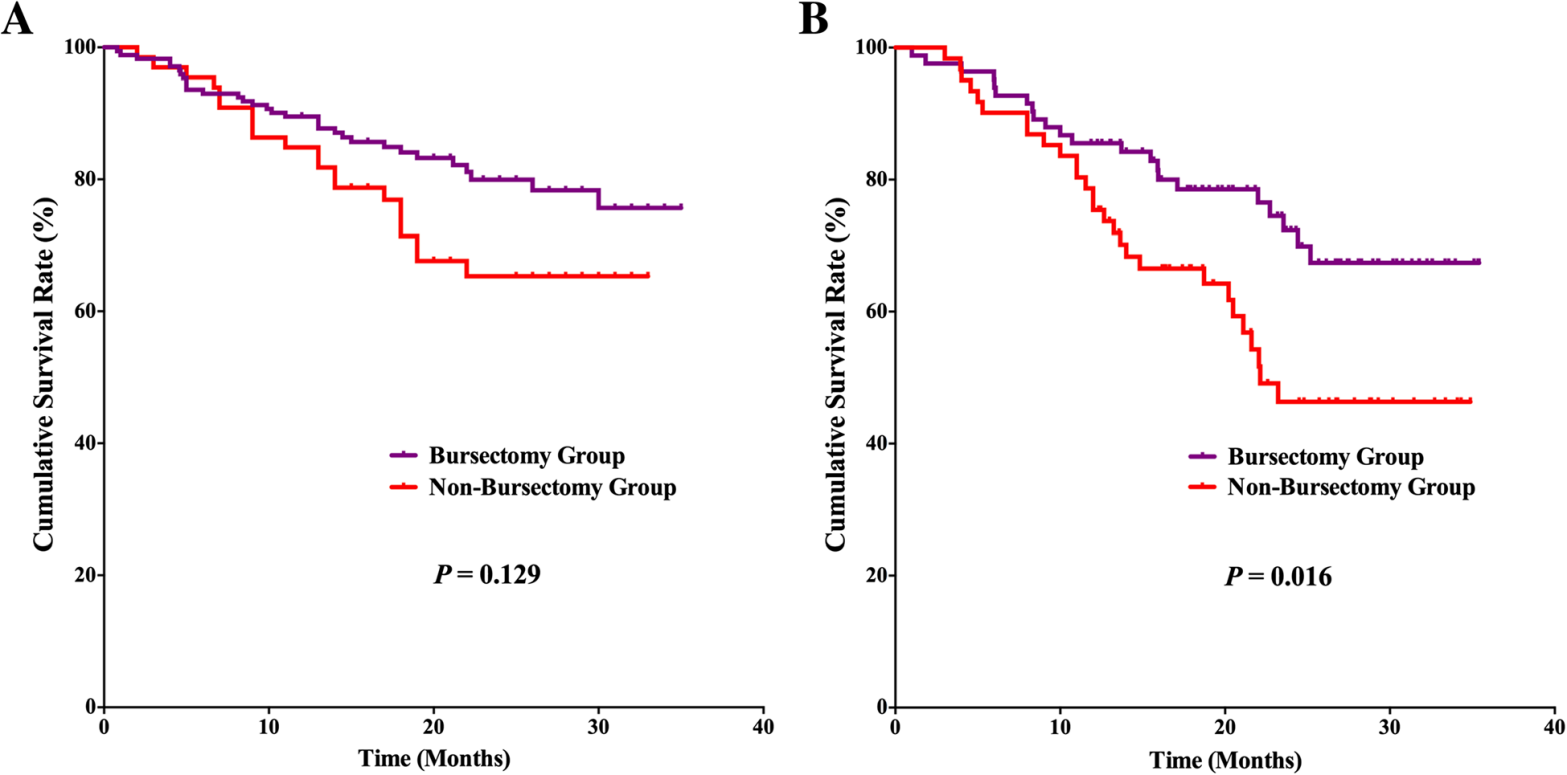
Survival curves among the bursectomy group and non-bursectomy group for patients who underwent distal gastrectomy (**a**) and total gastrectomy (**b**)

**Fig. 5:**
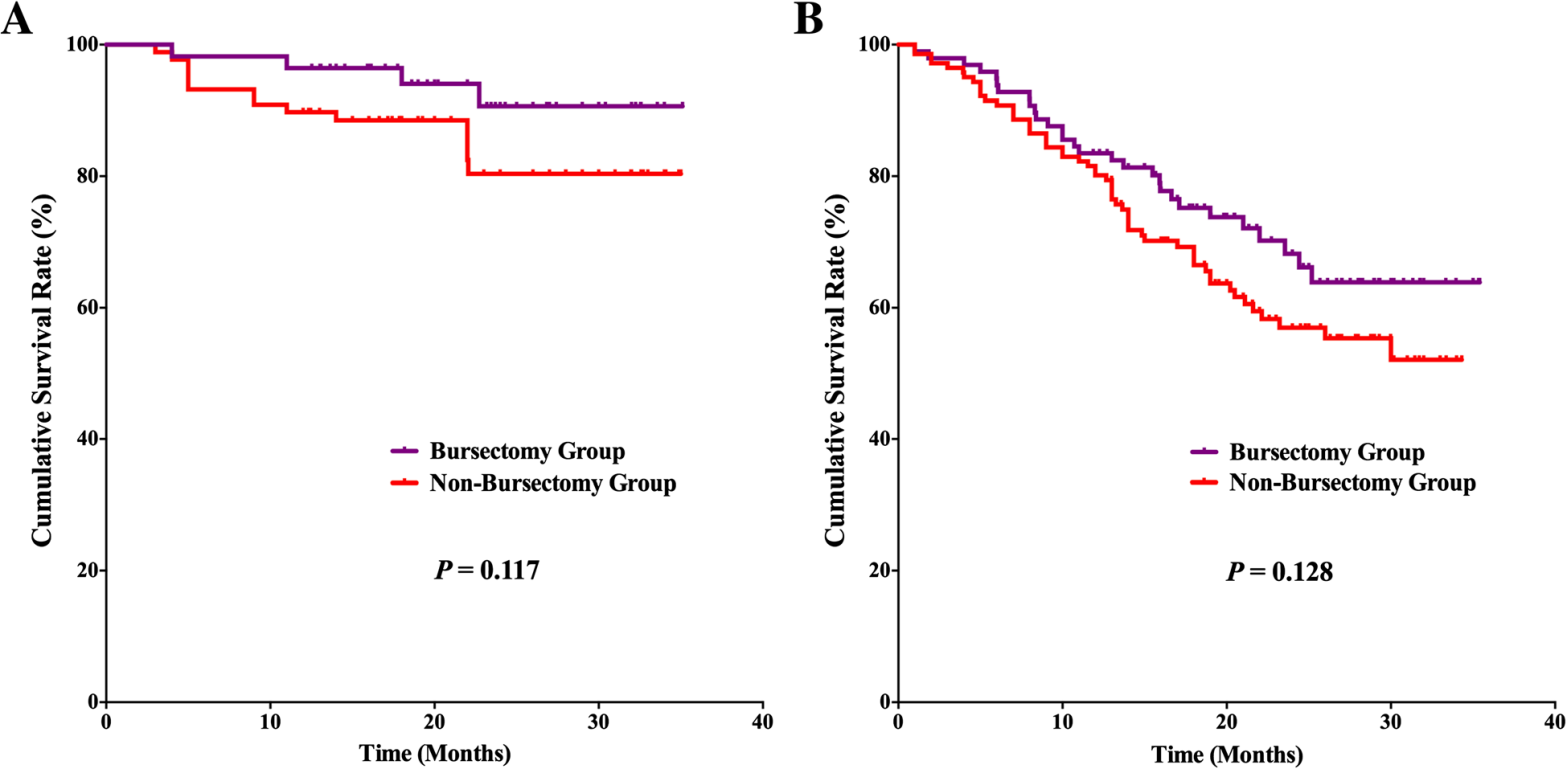
Survival curves among the bursectomy group and non-bursectomy group for patients with pT2-3 (**a**) stages and pT4a stages (**b**)

**Fig. 6:**
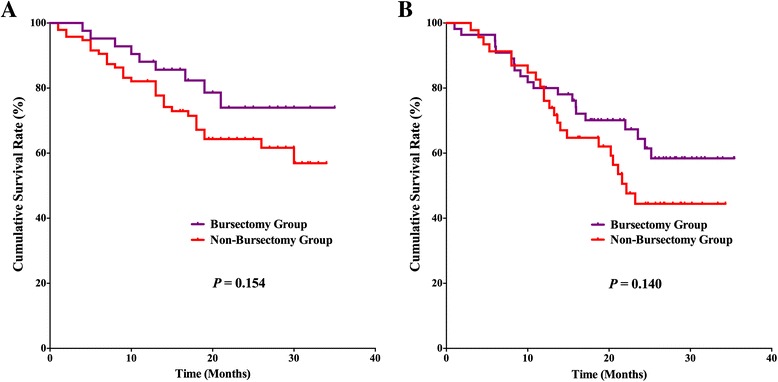
Survival curves among the bursectomy group and non-bursectomy group for patients with pT4a stage who underwent distal gastrectomy (**a**) and total gastrectomy (**b**)

For the postoperative follow-up, there were 382 patients with fully postoperative follow-up information and a 94.1 % follow-up rate, 20 (2–35) months median follow-up duration. Survival benefits can be observed in bursectomy group compared to non-bursectomy group, although without significant difference, *p* = 0.055 (Fig. 3). In the univariate survival analysis, the longitudinal location (*p* = 0.030), macroscopic type (*p* = 0.027), tumor size (*p* = 0.002), resection patterns (*p* = 0.012), pT stage (*p* < 0.001), and pN stage (*p* < 0.001) were prognostic risk factors for the overall survival. And in the multivariate analysis, bursectomy (without vs. with, *p* < 0.001), pT stage (pT2-3 stages vs. pT4 stage, *p* < 0.001), and pN stage (N0 vs. N3, *p* = 0.002) were independent prognostic risk factors for the overall survival (Table 4). Subgroup analyses were conducted in the resection patterns and pT stages. For those patients who underwent distal gastrectomy, the survival outcome was comparable between the two groups (*p* = 0.129), whereas the bursectomy group had better prognosis of patients who underwent total gastrectomy than patients in non-bursectomy group (*p* = 0.016) (Fig. 4a, b). There is no significant difference between the two groups for patients with pT2-3 stages (*p* = 0.117) and pT4 stages (*p* = 0.128) (Fig. 5a, b). And for patients with pT4 stage, although there exist differences in the survival curves, no significant differences are shown for patients who underwent distal gastrectomy (*p* = 0.154) and total gastrectomy (*p* = 0.160) (Fig. 6a, b)

**Table 4:** The univariate and multivariate survival analysis of all patients

	*p* value*	Multivariate HR (95 % CI)	*p* value**
Age	0.753		
<65 years vs. ≥65 years			
Gender	0.880		
Male vs. female			
Cross-sectional location	0.315		
Lesser vs. greater vs. anterior vs. posterior vs. circumferential involvement			
Longitudinal location	0.030		
U vs. M vs. L			
Differentiation grade	0.378		
Well-moderate vs. poor-undifferentiated			
Macroscopic type	0.027		
Type 1–2 vs. Type 3–4			
Tumor size	0.002		
<5 cm vs. ≥5 cm			
Resection patterns			
DG vs. TG	0.012		
Bursectomy	0.055		0.025
With vs. without		1:1.640 (1.064–2.528)	
T stage	<0.001	1	
pT2-3 vs. pT4		1:2.719 (1.615–4.579)	<0.001
N stage	<0.001		
N0		1	
N1		1.674 (0.812–3.450)	0.163
N2		1.936 (0.934–4.010)	0.075
N3		2.702 (1.461–4.997)	0.002

*Abbreviations*: *U* upper, M middle, *L* lower, *DG* distal gastrectomy, *TG* total gastrectomy

*Log-rank test; **Cox hazard model

## Discussion

In the 1960s, bursectomy is seen as an essential component of radical surgery for serosa-involved gastric adenocarcinomas in Japan. However, surgical safety and oncologic benefits are necessary factors in order to make sure bursectomy as a potential useful therapeutic procedure in the gastric cancer surgery according to the viewpoints today. By the results of the previous study, safety of bursectomy with D2 lymphadenectomy strongly hinges on the experience of surgeons [20]. With regard to the long-term survival outcomes, there was only one RCT that suggested that bursectomy had some survival benefits among the serosa-positive (pT3–T4) patients and without significant difference, the 3-year overall survival rate was 69.8 % for the bursectomy patients, in contrast to 50.2 % for the non-bursectomy group [9], and the 5-years follow-up results of this study existed similar results [21]. On the other hand, other studies had totally opposite results, and they did not find survival benefits of bursectomy when compared with non-bursectomy surgery [10–12]. Moreover, in China, more than half of gastric cancer patients were with advanced stage tumors. Therefore, we assessed the outcomes of advanced gastric cancer patients who underwent D2 gastrectomy with bursectomy or who are not in a single institute of China. In this study, we found that the postoperative complications rate was comparable between patients with or without bursectomy, and D2 gastrectomy with bursectomy had benefits in short-term overall survival outcomes when compared with non-bursectomy gastrectomy, especially for patients who underwent total gastrectomy.

Bursectomy is a complicated and technique-dependent procedure, which may increase the surgical duration and account for more blood loss during the operations. The previous Japanese randomized controlled trial found that bursectomy was associated with an additional 27 min surgical duration and an additional 125 ml intraoperative blood loss compared with non-bursectomy [20]. And another cohort study found that bursectomy procedure was associated with an additional 41 min operation times and an additional 65 ml intraoperative blood loss [22]. Also in our study, increasing of the surgical duration can be discovered in bursectomy group than the non-bursectomy group. The extra time consuming of the operations was mainly because of the dissection of the anterior of mesocolon of transverse and capsule of the pancreas. However, long surgical duration and high intraoperative blood loss do not mean the unsafety of the surgical bursectomy procedures although with the potential injury of the vessels of the mesocolon of transverse. Blouhos et al. [22] found that the postoperative morbidity rate was 19.4 % for patients with bursectomy, and Imamura et al. [20], in their study, presented that the overall morbidity rate was 14.3 % for both the bursectomy and non-bursectomy. In our study, the incidence of postoperative complications was comparable with the two groups (bursectomy group 23.3 % vs. non-bursectomy group 17.8 %, *p* = 0.179). Meanwhile, the grade of complications according to the Clavien–Dindo surgical complications classification [19] between the two groups was also comparable (*p* = 0.759). Therefore, despite the fact that the bursectomy is a time-consuming procedure, the D2 lymphadenectomy with gastrectomy plus bursectomy can be performed safely in high-volume experienced centers or by experienced surgeons [9].

Of the postoperative complications, in details, gastrointestinal surgeons are most concerned about the possible damage of the pancreas and potential trend increase the incidence of pancreatic fistula formation and postoperative intestinal obstruction. Injury to the pancreatic parenchyma may occur when dissecting the pancreatic capsule and lead to the probable incidence of pancreatic fistula. Previous study reported that subclinical pancreatic fistula could occur in up to 10 % of the patients with the resection of the pancreatic capsule [23]. And on the other hand, Imamura et al., in their study, observed that there was no difference in the incidence of pancreatic fistulas and amylase levels in the postoperative drainage fluid between the bursectomy group and non-bursectomy group [20]. They concluded that pancreatic fistula may not be caused by the dissection of the pancreatic capsule but owing to the lymphadenectomy of those lymph nodes adjacent to the pancreas parenchyma. Blouhos et al. reported that the incidence rate of pancreatic fistula is only 4.2 % (3/72) [22]. Generally, the resection of the pancreatic capsule is experienced cumulative procedures, and the experienced surgeons in an experienced center can rarely induce injury to the pancreas and reduce the potential incidence of pancreatic fistula [22].

Next, concerned postoperative adverse events are the possibility of intra-abdominal adhesion formation and the potential intra-abdominal intestinal adhesive obstruction. For the gastrectomy with bursectomy, adhesions to the mesocolon and pancreas may cause specific symptoms, such as delayed gastric emptying, afferent loop syndrome, or intestinal obstruction [20]. The early stage postoperative intestinal obstruction usually occurs in about 1–2 weeks after the operation. However, postoperative intestinal obstruction can occur at any point in time during the postoperative period. Therefore, short-term postoperative observation is not enough to fully evaluate the incidence of postoperative intestinal obstruction. Long-term follow-up and careful observation in a large sample size cohort study is expected to draw the conclusion about postoperative intestinal obstruction of bursectomy.

The most important purpose of total bursectomy is to eliminate the cancer cells for the prevention of potential peritoneal relapse and improve the survival outcomes. However, evidence of survival outcomes decides whether “bigger surgery is better.” For the tumor that penetrates the serosa of the stomach, some surgeons hold the attitude that bursectomy cannot eliminate all disseminated free cancer cells because the bursa omentalis is not a closed space and bursectomy is unlikely to improve overall survival in patients with subserosa or serosa invasive cancers [10]. Previous studies found that there was no statistical difference between the two groups in overall survival, and the bursectomy procedure was not recommended as a routinely procedure [5, 10–12, 24]. However, we also noticed that the interim analyses and the final reports of Osaka trial found that bursectomy surgery had some survival advantages in stage pT3-4 patients when compared with non-bursectomy surgery regarding the recurrence rate, recurrence type (peritoneal seeding recurrence) and the survival rates [9, 21]. Meanwhile, we noticed that there is no statistical difference between the recurrence rate and recurrence type between the two groups (Additional file 1: Table S1). Recurrence type is another important factor to evaluate the survival benefits for different resection strategies. These results maybe due to the short-term follow-up duration, which may not completely present the potential recurrence between two groups. There is no difference of survival outcomes between the two groups, but the bursectomy group had better curves than the non-bursectomy group (Fig. 1). The following may be the reasons for these results: the bursectomy may have some benefits in terms of prolonging the patients’ survival outcomes, but because of the sample size and limited follow-up duration result, the statistical difference did not appear. In view of these results, besides Japan and Korea, advanced stage gastric cancer account for about half of total gastric cancer patients [25–27], especially in China [28]. Therefore, a well-designed large sample RCTs with long-term study to find out the details operation indications of the bursectomy for advanced gastric cancer patients are expected.

Interestingly, we noticed that the number of harvested lymph nodes was significantly higher in the bursectomy group than the non-bursectomy group (40.6 ± 17.5 vs. 25.4 ± 9.9, respectively, *p* < 0.001). Meanwhile, the number of positive lymph nodes was also higher in the bursectomy group than the non-bursectomy group (7.5 ± 8.7 vs. 5.9 ± 6.4, respectively, *p* = 0.045). Lymph node dissection strategy is one of the hottest topics regarding gastric cancer surgery. On one hand, because of the limitation of the retrospective study, the potential selection bias may be one reason, which results to the different lymph node results between the two groups. Although, we only include those patients who underwent surgical treatment by surgeons of gastric cancer surgical treatment team in our department, which aims to dimension the potential bias coming from operators. On the other hand, Greece scholar Blouhos et al. presented right side bursectomy as an access plane for completely en bloc dissection of lymph nodes [29]. This may be another reason for the different lymph node result between the two groups. It is certain that both the Western and Eastern guidelines recommended at least 16 lymph nodes examined for gastric cancer surgery in the N stage determination [30]. In our study, the non-bursectomy dissected 25.4 ± 9.9 lymph nodes, which is higher than the recommendation of the guidelines. Also, some study reported that the number of lymph node dissection is closely related with the prognosis of patients [31–34]. Previous study identified that for stage N2–N3 gastric cancer patients, harvesting at least 25 lymph nodes may represent a superior cutoff for radical gastrectomy and could yield better survival outcomes [35]. Therefore, regardless of the fact that there existed a significant difference in the number of lymph nodes between the two groups, the number of examined lymph nodes in non-bursectomy is higher than the requirements of the present gastric cancer classification.

Meanwhile, different from the previous study, we included those patients who underwent total gastrectomy. Although, the results of this study found that there was no significant difference in the short-term survival outcomes between bursectomy and non-bursectomy groups. For those patients who underwent total gastrectomy, the survival benefit of the bursectomy has appeared. Note that despite that there is no significant difference in survival outcomes for distal gastrectomy patients or pT4 stage patients, the survival curves had demonstrated the separated trending between the two surgical strategies. As the previous description, sample size and short-term follow-up duration may be the reasons for these survival results. Also, upper third gastric cancers usually had poor tumor characteristics and survival outcomes than lower third gastric cancers [36]. Besides, those lower third gastric cancers that underwent total gastrectomy are generally due to the poor tumor characteristics. These may be the main reasons why there exists a statistical difference between the two groups in total gastrectomy and not in distal gastrectomy. Despite the fact that this study set ups subgroup analysis in the resection patterns and conducts the multivariate survival analysis which identified that bursectomy or not is one of the independent prognostic risk factors, the potential selection bias of the retrospective study was existing and which must be emphasized here again. Regardless of the fact that previous and present evidence did not strongly prove that the bursectomy should be routinely performed, this technique-dependent procedure may be existing survival benefits in gastric cancer patients with specific characteristics, such as T stages, tumor locations, or other characteristics. Thus, well-designed RCTs are expected to clear these.

## Conclusions

As a highly technique-dependent procedure, bursectomy resulted in longer surgical duration than non-bursectomy surgery. Experienced surgeons could safely perform D2 gastrectomy with bursectomy and the postoperative complications not increased. Whether the bursectomy has survival benefits for all or special characteristics gastric cancer patient and surgical indications of bursectomy surgery is pending.

## Additional file

Additional file 1: Table S1. Tumor recurrence between the two groups.

## Abbreviations

LNs: lymph nodes
RCT: randomized controlled trial
JGCA: Japanese Gastric Cancer Association
UICC: Union for International Cancer Control
TNM: tumor-node-metastasis
JCOG: Japan Clinical Oncology Group
