# Supplementary material for: Bursectomy and non-bursectomy D2 gastrectomy for advanced gastric cancer, initial experience from a single institution in China
Source: World J Surg Oncol. 2015 Dec 8;13:332. doi: 10.1186/s12957-015-0744-x (PMC4672481; doi:10.1186/s12957-015-0744-x)
Supplement: Additional file 1: Table S1. — Tumor recurrence between the two groups. [file 12957_2015_744_MOESM1_ESM.doc]

**Additional file 1**

**Table S1. Tumor recurrence between the two groups**

|  | | | |
| --- | --- | --- | --- |
|  | **Non-Bursectomy Group**  **N=247 (%)** | **Bursectomy Group**  **N=159 (%)** | **P Value** |
|  |  |  |  |
| **Tumor Recurrence** | 35 (14.2) | 25 (15.7) | 0.667 |
| **Recurrence Type** |  |  | 0.492 |
| Locoregional recurrence | 3 | 4 |  |
| Peritoneal recurrence | 18 | 10 |  |
| Hematogenous recurrence | 6 | 7 |  |
| Distal lymph nodes | 3 | 3 |  |
| Multi-site recurrence | 5 | 1 |  |
